# Values of prognostic nutritional index for predicting Kawasaki disease: a systematic review and meta-analysis

**DOI:** 10.3389/fnut.2024.1305775

**Published:** 2024-02-02

**Authors:** Xiaoling Zhong, Yu Xie, Hui Wang, Guihua Chen, Taoyi Yang, Jiang Xie

**Affiliations:** ^1^Department of Pediatrics, The Third People’s Hospital of Chengdu, The Affiliated Hospital of Southwest Jiaotong University, Chengdu, China; ^2^Jinniu District Maternity and Child Health Hospital of Chengdu, Chengdu, China

**Keywords:** prognostic nutritional index, Kawasaki disease, coronary artery lesion, intravenous immunoglobulin resistance, meta-analysis

## Abstract

**Objective:**

This systematic review and meta-analysis aimed to evaluate the relationship between the prognostic nutritional index (PNI) and intravenous immunoglobulin (IVIG) resistance and coronary artery lesion (CAL) in Kawasaki disease (KD).

**Methods:**

The relevant literature was searched on PubMed, Embase, Cochrane Library, Web of Science, and Google Scholar up to August 5, 2023. A pooled sensitivity, specificity, positive likelihood ratio (PLR), negative likelihood ratio (NLR), diagnostic odds ratio (DOR), and area under curve (AUC) were calculated to assess the predicted values of PNI in KD patients with IVIG resistance and CAL.

**Results:**

A total of 8 articles containing 10 studies involving 7,047 participants were included. The pooled results revealed a pooled sensitivity of 0.44 (0.25–0.65), a pooled specificity of 0.87 (0.73–0.94), a pooled PLR of 3.4 (2.0–5.9), a pooled NLR of 0.65 (0.48–0.87), a pooled DOR of 5.26 (2.76–10.02), and a pooled AUC of 0.75 (0.71–0.78) in the diagnosis of KD with CAL. The pooled results suggested that a pooled sensitivity was 0.69 (0.60–0.77), specificity was 0.76 (0.69–0.82), PLR was 2.9 (2.1–4.1), NLR was 0.40 (0.29–0.56), DOR was 7.27 (3.89–13.59), and AUC was 0.79 (0.75–0.82) in the diagnosis of KD with IVIG resistance. The combined results revealed the pooled sensitivity was 0.63 (0.58–0.67), specificity was 0.82 (0.80–0.83), PLR was 3.09 (1.06–8.98), NLR was 0.38 (0.07–2.02), DOR was 8.23 (0.81–83.16) in differentiating KD from febrile patients. These findings demonstrated low sensitivity and relatively high specificity of PNI for KD, KD-CAL, and IVIG-resistant KD.

**Conclusion:**

In conclusion, this study was the first systematic review and meta-analysis of the diagnostic value of PNI in KD with IVIG resistance and CAL. The results suggested that PNI could be used as biomarkers for distinguish KD, KD with CAL, and KD with IVIG resistance.

## 1 Introduction

Kawasaki disease (KD) is an acute febrile disease of unknown etiology, and the main symptom is systemic vasculitis, which easily affects the coronary artery (1). Moreover, the latest epidemiological survey discovered that KD has a high incidence in East Asia, especially in Japan and China, with a relatively average distribution in other places (2). The most serious complication in KD patients is coronary artery lesion (CAL), which lead to coronary artery aneurysm (CAA), thrombosis, myocardial infarction, heart failure and even sudden death and are the main cause of childhood acquired heart disease worldwide (3, 4). Intravenous immunoglobulin (IVIG) has been proven to reduce the development of CAL in patients with KD to approximately 4%, but approximately 10–20% of KD patients are resistant to IVIG, and some studies have shown that IVIG-resistant KD patients have a higher risk of CAL (5–7). The diagnosis of KD is currently mostly based on the clinical manifestations of patients, which directly leads to the misdiagnosis or delayed treatment of some patients with atypical clinical symptoms and increases the occurrence of CAL. Therefore, studies of KD have focused on exploring the underlying biomarkers to diagnose or predict CAL and IVIG resistance in KD patients.

The prognostic nutritional index (PNI) was first reported in 1983 and initially used to evaluate nutritional status after gastrointestinal surgery (8). For several decades, PNI has developed rapidly and has been regarded as an important indicator to evaluate and predict the prognosis of various malignant tumors, reflecting the nutritional and immune status of the body, as well as predicting the outcome of cardiac surgery and coronary artery disease in pediatric patients (9–13). PNI is the combination of serum albumin levels and peripheral blood lymphocyte count and is thought to embody the inflammatory condition of patients to a certain extent (14). Systemic inflammation plays a significant role in the occurrence and development of KD.

In recent years, some studies have suggested that PNI could predict IVIG resistance and CAL in patients with KD (15–18). However, there are no consistent conclusions about the diagnostic accuracy of PNI in KD patients with IVIG resistance and CAL. In addition, no systematic reviews have attempted to summarize the available evidence. Therefore, the main purpose of the current systematic review and meta-analysis is to summarize the value of PNI in predicting CAL and IVIG resistance in KD patients.

## 2 Methods

This systematic review/meta-analysis was reported in accordance with the Preferred Reporting Items for Systematic Reviews and Meta-Analyses: The PRISMA. Statement (19).

### 2.1 Literature retrieval

We performed online searches in the following databases: PubMed, Embase, Cochrane Library, Web of Science, and Google Scholar. The search date was from inception to August 05, 2023. No language was restricted during the search. The following search terms were used for literature research: “mucocutaneous lymph node syndrome” or “Kawasaki syndrome” or “lymph node syndrome, mucocutaneous” or “Kawasaki disease,” “prognostic nutritional index” or “PNI” or “Prognostic Nutritional Indices.” These search terms were combined with Boolean operators “OR” and “AND.” In addition, some research references were searched manually. This meta-analysis has been registered on the International Platform of Registered Systematic Review and Meta-analysis Protocols (INPLASY2023100003).

### 2.2 Inclusion and exclusion criteria

The criteria for inclusion were as follows:

1. Studies about the diagnostic performance of PNI in KD patients.
2. There was no limitation on the sample size or the treatment regimen of KD in the included studies.

The exclusion criteria were as follows:

1. Irrelevant study topic.
2. Duplicate studies, animal and experimental studies, reviews, conference articles, expert opinions, abstracts, or case reports without controls.
3. There was not enough data for meta-analysis.

### 2.3 Literature screening and data extraction

Two independent reviewers conducted the screening of literature, data extraction, and cross-verification. Initially, duplicate records were removed, followed by the exclusion of studies unrelated to the research topic through scanning titles and abstracts. Finally, full-text reading was employed to determine eligible inclusion of literature. Any disagreements were resolved through consensus. Data were extracted using a customized data extraction form. The data extraction primarily included (1) information about the included studies, such as titles, first author, and publication year; (2) basic characteristics of the included study subjects (sample size, age, sex, and diagnostic criteria); (3) study design type; (4) outcome measures, including different cutoff values for the PNI, sensitivity, specificity, and AUC.

### 2.4 Assessment of quality in included studies

Quality assessment and cross-verification of the results were conducted by two independent reviewers. Any discrepancies were resolved by consensus. The assessment of bias risk utilized the Quality Assessment of Diagnostic Accuracy Studies-2 (QUADAS-2) tool, which was recommended by the Cochrane Library Handbook for assessing diagnostic research (20).

### 2.5 Statistical analysis

Data analysis for this study was conducted using Stata 12.0, RevMan 5.4, and Meta-disc 1.4 software. Cochran’s *Q*-test and *I*^2^-test assessed the heterogeneity and were used to determine the effect model. If there was no significant statistical heterogeneity among the study results (*I*^2^ < 50%, *p* ≥ 0.05), a fixed-effects model was employed for the meta-analysis. In cases where there was statistical heterogeneity among the study results, a random-effects model was used for the meta-analysis (*I*^2^ ≥ 50%, *p* < 0.05). Studies exhibiting significant clinical heterogeneity were subjected to sensitivity analysis. Deek’s funnel plot was used to evaluate publication bias. *p* < 0.05 was considered significant.

## 3 Results

### 3.1 Literature screening process and results

**Figure 1:**
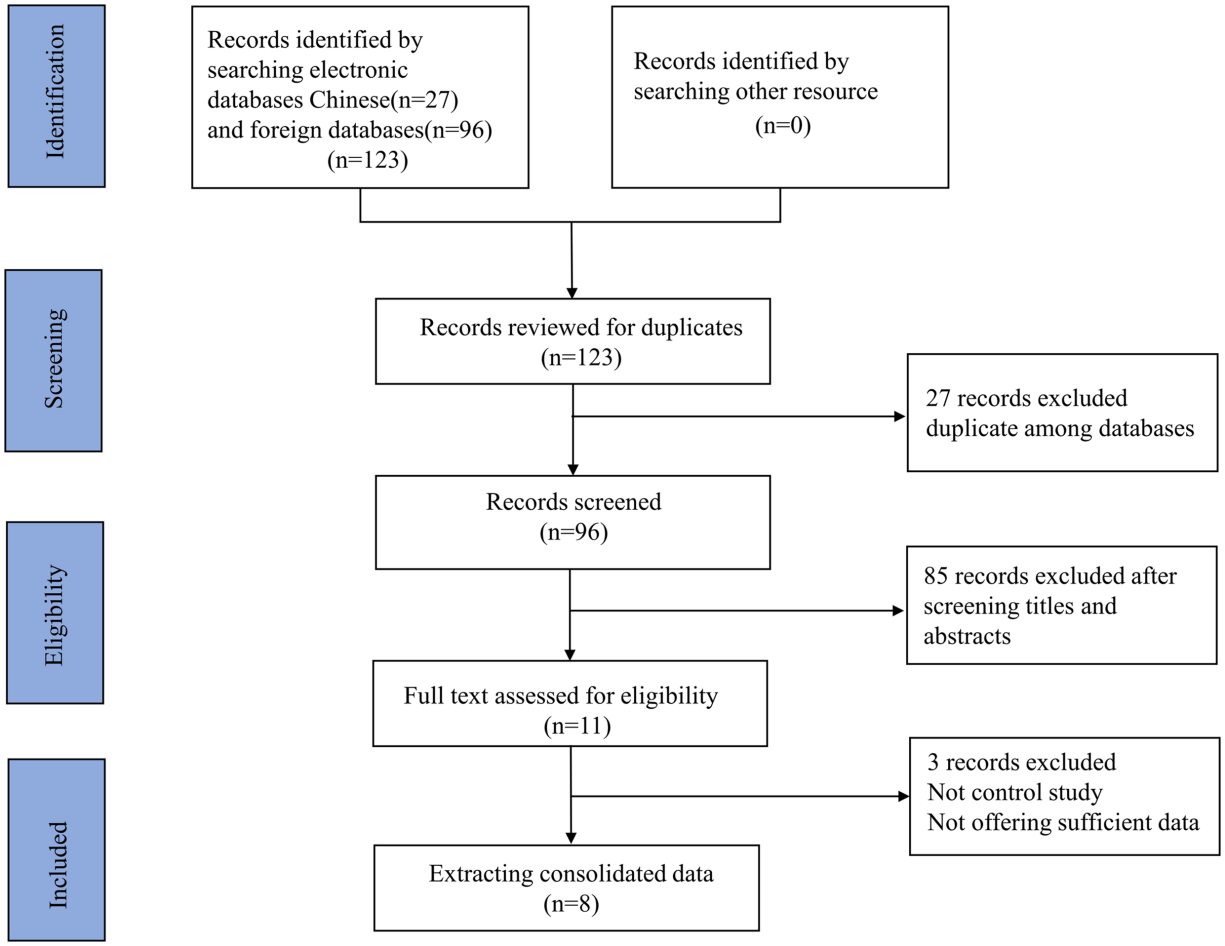
A flow diagram demonstrating the study selection process.

The screening process is displayed in Figure 1. The initial screening yielded 123 relevant articles, including PubMed (*n* = 10), Embase (*n* = 8), Cochrane Library (*n* = 0), Web of Science (*n* = 9), and Google Scholar (*n* = 96). No other study was retrieved by manual search. Twenty-seven duplicate articles were excluded, leaving 96 studies for subsequent screening. After reviewing the titles and abstracts, 85 articles were excluded. Subsequently, a total of 11 full-text articles were reviewed. Finally, 8 articles containing 10 studies were eligible. Of these studies, a total of 4 studies assessed the value of PNI in differentiating KD-CAL from KD-NCAL. A total of 4 studies focused on the value of PNI in differentiating KD-IVIG from KD-responsive patients. Only 2 studies assessed the diagnostic value of the PNI in differentiating KD patients from febrile controls.

### 3.2 Basic characteristics of included studies

The summarized characteristics of the included studies are presented in Table 1. From 2020 to 2023, a total of 8 articles containing 10 studies were eligible (15–18, 21–24). The majority of these studies were conducted in mainland China (8/10), one study from Taiwan, China, and one from Turkey. Most studies (8/10) employed the American Heart Association (AHA) criteria as the gold standard for the diagnosis of KD, while only 2 studies used a combination of the AHA and Japanese Cardiology Society (JCS) criteria for diagnosis. Four studies applied the AHA criteria for diagnosing CAA, and 4 studies considered fever persistence beyond 36 h after IVIG administration as the diagnostic criterion for IVIG resistance. In addition, Li et al. also recommended recurrent fever ≥38°C and recrudescence of one or more of the initial symptoms as criteria for IVIG resistance (15). Treatment regimens across the studies were not entirely consistent, as shown in Table 1. The most commonly used treatment regimen was IVIG (2 g/kg given as a single intravenous infusion) in combination with aspirin (30–50 mg/kg/day). Only two studies were prospective, while the majority of studies (8/10) were retrospective. Only one study was conducted at multiple centers, while eight studies were single-center studies and one was unclear. The PNI cutoff values ranged from 31.5 to 55.24. There were a total of 470 cases of KD-CAL patients, 1,035 cases of KD-NCAL patients, 370 cases of IVIG-resistant patients, and 2,668 cases of IVIG—responsive patients. Furthermore, in a study evaluating the value of the PNI in distinguishing between KD and febrile patients, 406 KD patients and 2098 febrile patients were included (Tables 1, 2).

**Table 1:** The primary characteristics of the included studies.

References	Country	Type of study	Control	Diagnosis of KD	Diagnosis of CAL	Diagnosis of IVIG-resistant KD	Time frame	Single-center/multi-center	Treatment
Tai et al. (21)	Taiwan, China	Retrospective	NCAL	AHA	AHA	N/R	2012–2016	Single center	N/R
Liu et al. (16)	China	Prospective	NCAL	AHA	AHA	Recurrent or persistent fever or other clinical signs of KD for at least 36 h after initial IVIG, the second IVIG (2 g/kg given as a single intravenous infusion) was given	2015–2019	Single-center	IVIG (1 g/kg/day) plus oral aspirin (30–50 mg/kg/day)
Yalcinkaya et al. (17)	Turkey	Retrospective	NCAL	AHA	AHA	A persistent fever lasting > 36 h after IVIG completion or recurrent fever associated with KD symptoms after an febrile period	2008–2019	N/R	IVIG (2 g/kg) as a single intravenous infusion plus oral aspirin (60 mg/kg/day) within the first 10 days of illness from the beginning of fever
Liu et al. (18)	China	Retrospective	NCAL	AHA	AHA	Patients showed symptoms of persistent or recrudescent fever (axillary or rectal temperatures of ≥37.5°C and ≥38.0°C, respectively) for ≥36 h, but ≤7 days after receiving the initial IVIG infusion (2 g/kg), and received a second dose of gamma globulin therapy	2013–2020	Single-center	IVIG (2 g/kg) and aspirin
Li et al. (15)	China	Retrospective	IVIG-sensitive	AHA	AHA	Recurrent fever≥38°C and recrudescence of one or more of the initial symptoms, or persistent fever for at least 36 h after completion of initial IVIG infusion	2013–2020	Multi-center	IVIG(1 g/kg/day) plus oral aspirin (30–50 mg/kg/day)
Li et al. (22)	China	Retrospective	IVIG-sensitive	AHA and JCS	N/R	Having fever or re-fever after 36 h of initial IVIG treatment	2018–2019	Single-center	IVIG (2 g/kg) plus oral aspirin (30-50 mg/kg/day)
Li et al. (22)	China	Retrospective	IVIG-sensitive	AHA and JCS	N/R	Having fever or re-fever after 36 h of initial IVIG treatment	2008–2019	Single-center	IVIG (2 g/kg) plus oral aspirin (30–50 mg/kg/day)
Liu et al. (16)	China	Prospective	IVIG-sensitive	AHA	AHA	Recurrent or persistent fever or other clinical signs of KD for at least 36 h after initial IVIG, the second IVIG (2 g/kg given as a single intravenous infusion) was given	2015–2019	Single-center	IVIG (2 g/kg) given as a single intravenous infusion plus aspirin (30–50 mg/kg/day)
Huang et al. (23)	China	Retrospective	Febrile control group	AHA	N/R	IVIG responsiveness was defined as the abatement of fever within 48 h after completing IVIG treatment and no return of fever (>38°C) for at least 7 days after with a marked improvement or normalization of the comorbid signs of inflammation	2016–2019	Single-center	N/R
Guo et al. (24)	China	Retrospective	Febrile control group	AHA	N/R	N/R	2018–2022	Single-center	N/R

N/R, not report.

**Table 2:** Main characteristics of the included studies.

References	Case (male/female)	Age of case	Control (male/female)	Age of control	PNI cut-off value	AUC	95%CI	Sensitivity	Specificity	TP	FP	FN	TN
Tai et al. (21)	149	1.14 y	126	1.31 y	55.24	0.588	0.513–0.663	0.500	0.678	75	41	74	85
Liu et al. (16)	86	N/R	669	N/R	38.6	0.556	0.488–0.624	0.174	0.957	15	29	71	640
Yalcinkaya et al. (17)	39	N/R	95	N/R	31.5	0.834	0.761–0.908	0.744	0.811	29	18	10	77
Liu et al. (18)	CAD 116(88/28) CAA 80(65/15)	CAD 23.00 (13.25, 34.00) m CAA 21.00 (11.25, 36.00) m	145 (87/58)	27.00 (16.00,48.50) m	40.2	0.708	0.569–0.847	0.410	0.920	80	12	116	133
Li et al. (15)	187	19 m	1070	N/R	49.5	0.707	0.681–0.732	0.6795	0.6601	127	364	60	706
Li et al. (22)	47 (30/17)	22.0 (12.0, 39.0) m	588 (388/200)	23.0 (12.0, 42.0) m	52.4	0.850	0.798–0.901	0.809	0.776	38	132	9	456
Li et al. (22)	33 (26/7)	34.0 (14.5, 53.0) m	358 (226/132)	22.0 (12.0, 39.0) m	52.4	0.839	N/R	0.727	0.849	24	54	9	304
Liu et al. (16)	103 (54/49)	2.4 (1.3–4.7) y	652 (377/275)	2.1 (1.1–3.6) y	47.8	0.718	0.684–0.750	0.573	0.753	59	161	44	491
Huang et al. (23)	216 (118/98)	21 m	494 (304/190)	21 m	52.03	0.630	0.584–0.677	0.434	0.767	94	115	122	379
Guo et al. (24)	190 (121/69)	1.00 (1.00–2.00) y	1604 (968/636)	1.00 (1.00–2.00) y	N/R	0.921	0.900–0.942	0.844	0.834	160	266	30	1338

TP, true positive; FP, false positive; FN, false negative; TN, true negative; CAD, coronary artery dilatation.

### 3.3 Assessment of quality

**Figure 2:**
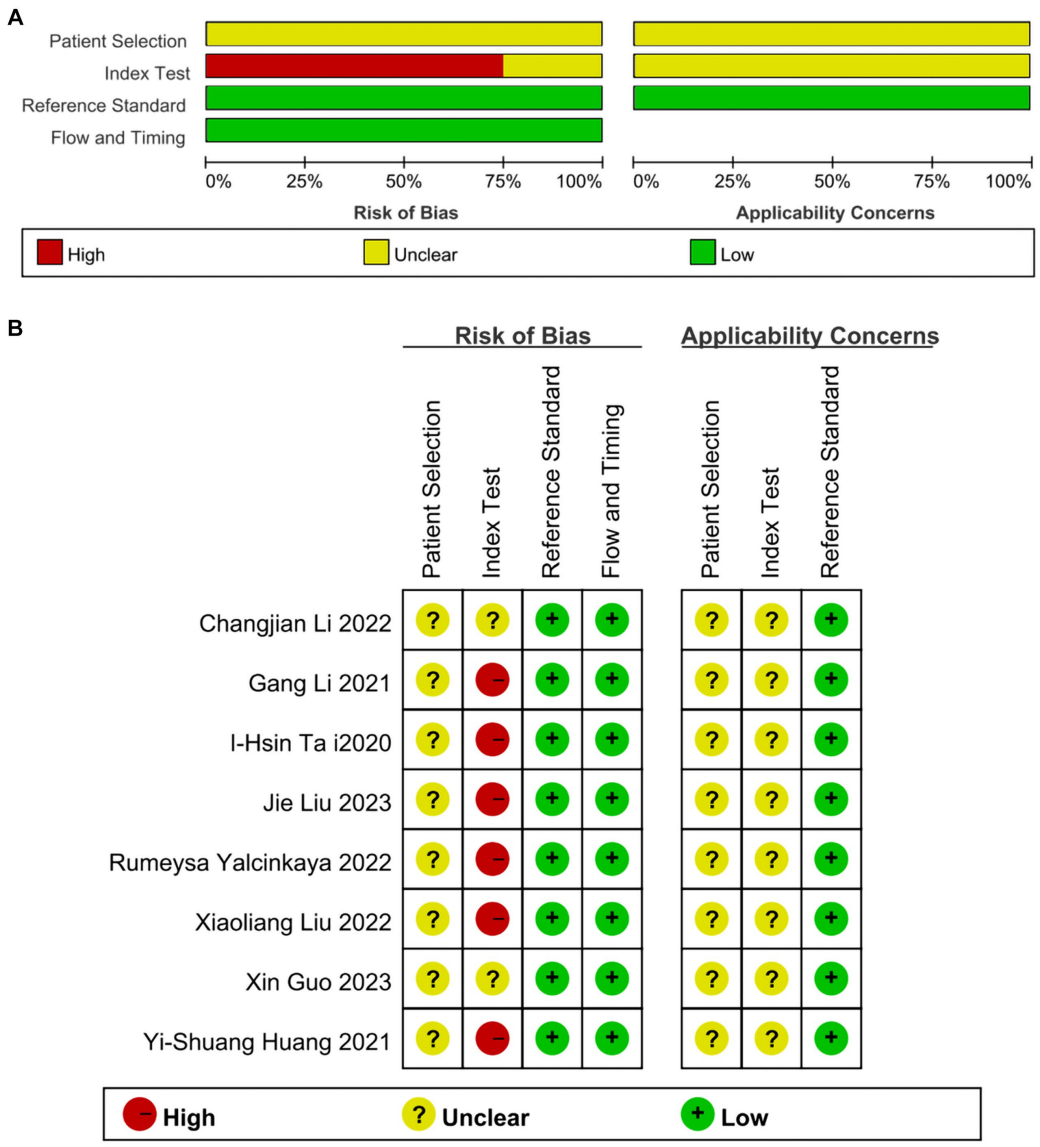
**(A)** Risk of bias and applicability concerns graph: reviews the judgments of the author about each domain presented as percentages across included studies. **(B)** Risk of bias and applicability concerns summary: reviews judgments of the author about each domain for each included study.

The QUADAS-2 assessment revealed that all included studies demonstrated a relatively low risk of bias in the reference standard, flow, and timing domains. However, all studies exhibited a moderate to high risk of bias in patient selection and index test domains. All included studies were considered to have low risk in terms of applicability within the reference standard, whereas for patient selection and index test, they were deemed “unclear.” Most studies were not primarily designed as diagnostic accuracy studies, and some items were not applicable. Thus, the researchers stated it as “unclear.” The results are depicted in Figures 2A,B.

### 3.4 Data synthesis and analysis

#### 3.4.1 The diagnostic performance of PNI for KD-CAL

**Figure 3:**
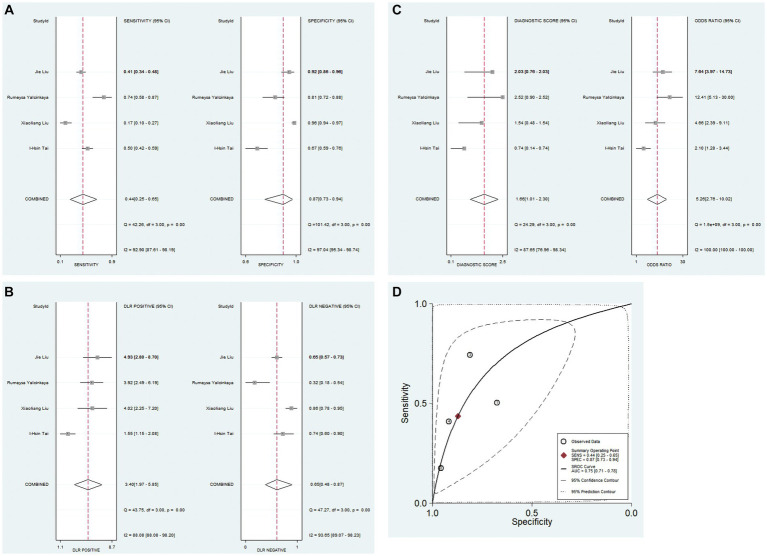
Pooled results of the studies on PNI used in the diagnosis for KD-CAL among 4 studies included in the meta-analysis. **(A)** Pooled sensitivity and specificity; **(B)** pooled PLR and NLR; **(C)** pooled DOR; **(D)** SROC.

A total of 4 studies analyzed the diagnostic value of PNI for KD with CAL. The combined results indicated significant heterogeneity (*I*^2^ = 82.0%, *p* < 0.05), and the random-effects model was used. The pooled results revealed a pooled sensitivity of 0.44 (0.25–0.65), a pooled specificity of 0.87 (0.73–0.94), a pooled positive likelihood ratio (PLR) of 3.4 (2.0–5.9), a pooled negative likelihood ratio (NLR) of 0.65 (0.48–0.87), a pooled diagnostic odds ratio (DOR) of 5.26 (2.76–10.02), and a pooled area under curve (AUC) of 0.75 (0.71–0.78). These findings suggested that PNI exhibited low sensitivity and relatively high specificity in the diagnosis of KD with CAL (Figures 3A–D).

#### 3.4.2 The diagnostic performance of PNI for IVIG-resistant KD patients

**Figure 4:**
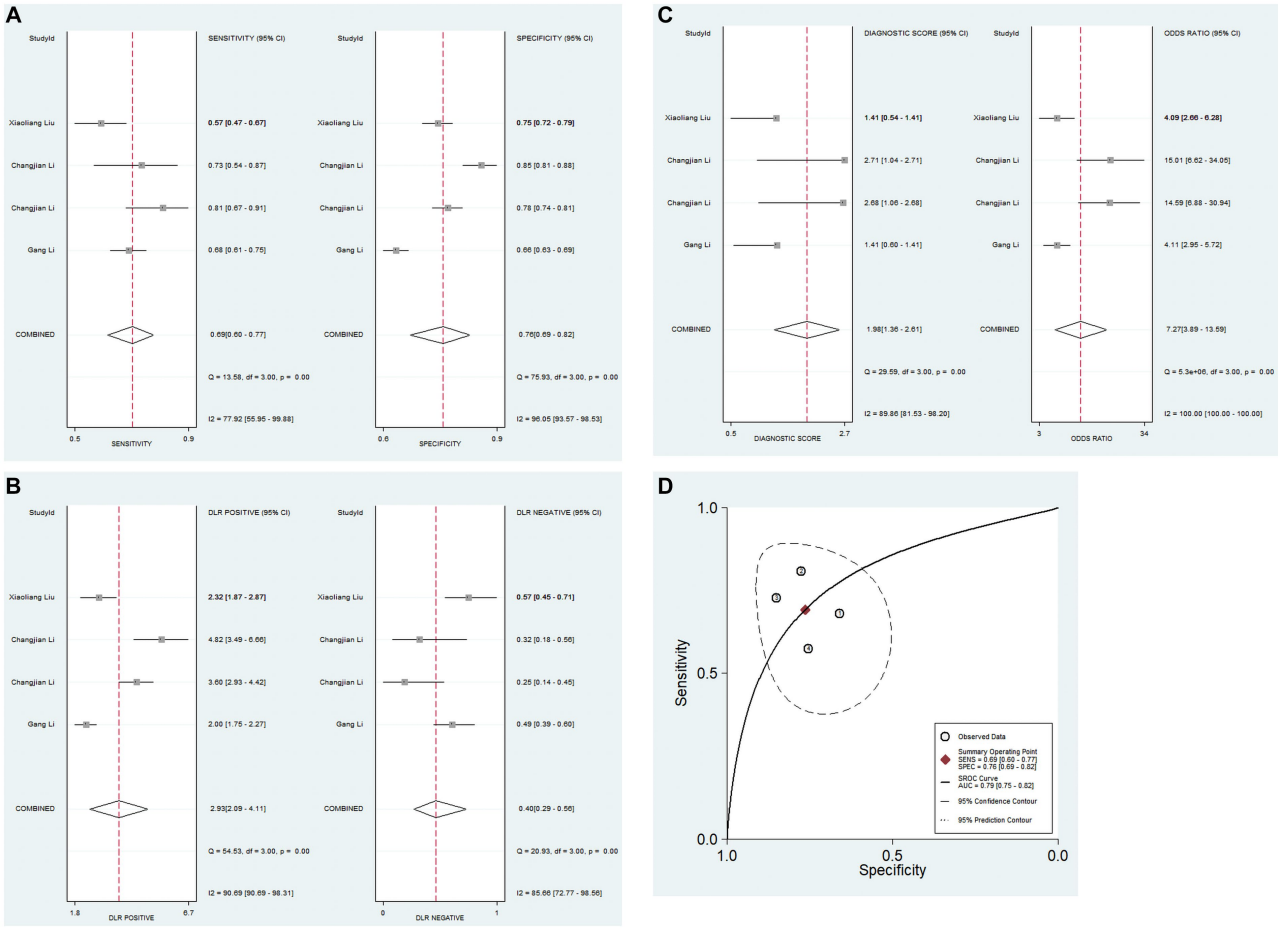
Pooled results of the studies on PNI used in the diagnosis for IVIG-resistant KD patients among 4 studies included in the meta-analysis. **(A)** Pooled sensitivity and specificity; **(B)** pooled PLR and NLR; **(C)** pooled DOR; **(D)** SROC.

A total of 4 studies analyzed the diagnostic value of PNI for IVIG resistance in KD. Due to significant heterogeneity among the studies (*I*^2^ = 82.2%, *p* < 0.05), the random-effects model was used. The pooled results revealed that the pooled sensitivity was 0.69 (0.60–0.77), pooled specificity was 0.76 (0.69–0.82), PLR was 2.9 (2.1–4.1), NLR was 0.40 (0.29–0.56), DOR was 7.27 (3.89–13.59), and AUC was 0.79 (0.75–0.82). These findings suggested that PNI demonstrated low sensitivity and relatively high specificity for IVIG resistance in KD patients (Figures 4A–D).

#### 3.4.3 The diagnostic performance of PNI in differentiating KD from febrile patients

**Figure 5:**
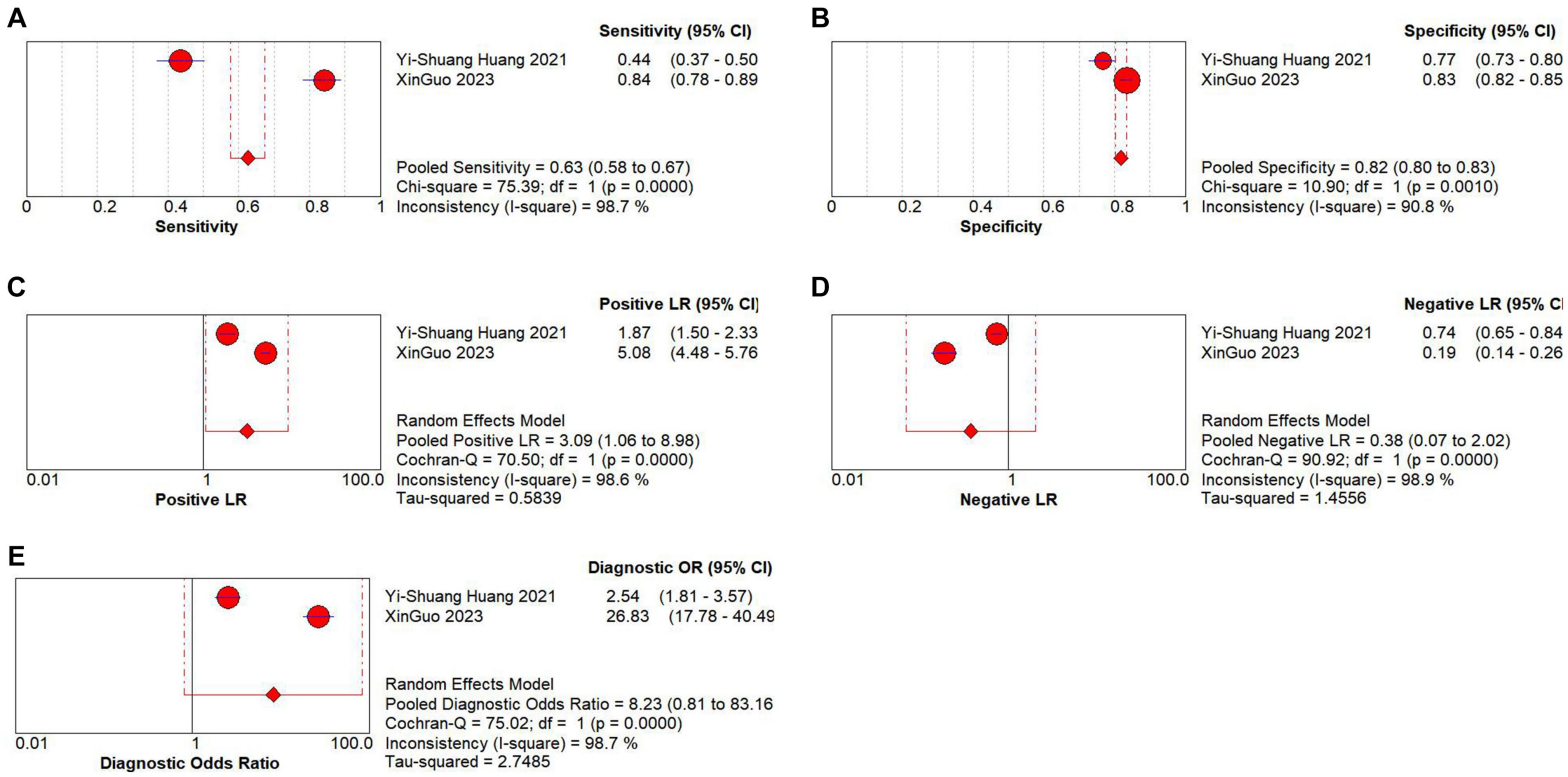
Pooled results of the studies on PNI used in the diagnosis for differentiating KD patients from febrile illness among 2 studies included in the meta-analysis. **(A)** Pooled sensitivity; **(B)** pooled specificity; **(C)** pooled PLR; **(D)** Pooled NLR; **(E)** pooled DOR.

Only 2 studies have explored the diagnostic value of PNI in distinguishing KD from other febrile illnesses. There was evident heterogeneity across these studies (*I*^2^ = 98.7%, *p* < 0.05), and the random-effects model was used to calculate the pooled results. The combined results revealed that the pooled sensitivity was 0.63 (0.58–0.67), the specificity was 0.82 (0.80–0.83), the PLR was 3.09 (1.06–8.98), the NLR was 0.38 (0.07–2.02), and the DOR was 8.23 (0.81–83.16). These results suggested that PNI exhibited low sensitivity and relatively high specificity in the context of KD diagnosis, and the current evidence indicated that PNI has low sensitivity and relatively high specificity in differentiating KD from other febrile illnesses (Figures 5A–E).

### 3.5 Sensitivity analysis

**Figure 6:**
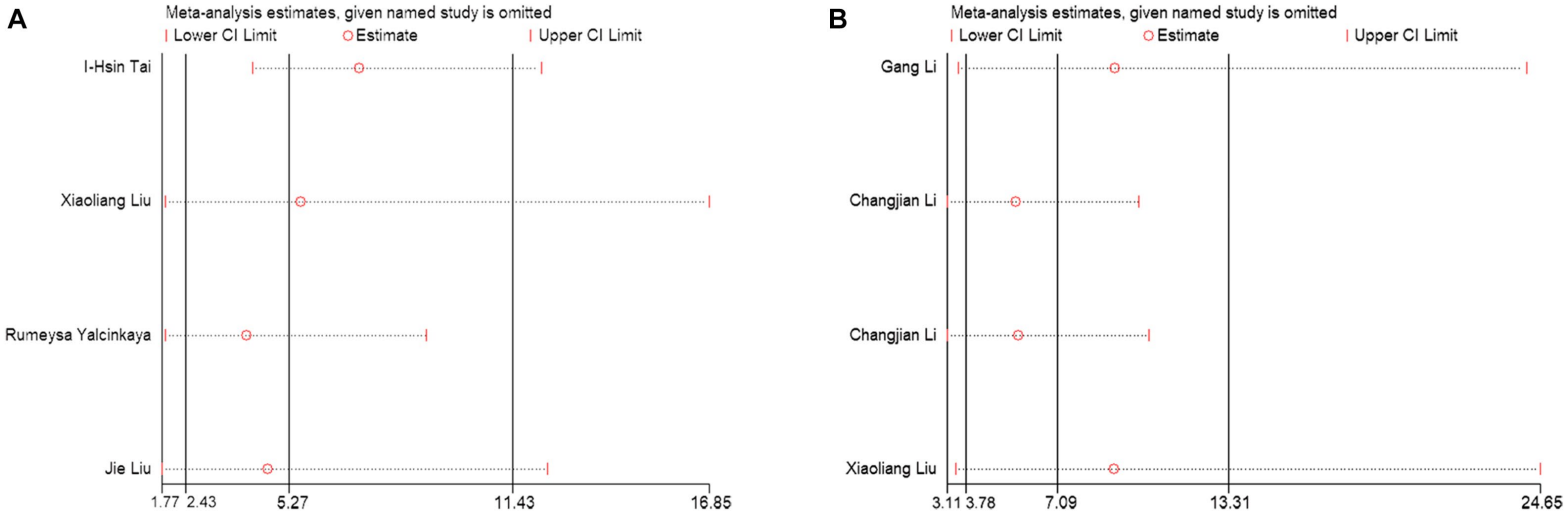
Sensitivity analysis of the results of the meta-analysis. **(A)** PNI diagnosis for KD-CAL; **(B)** PNI differentiating IVIG-resistant KD patients from IVIG-responsive KD patients.

Sensitivity analysis was used to assess the contribution of each study to the combined estimate by systematically excluding one study at a time and recalculating the effect sizes for the remaining studies. Our sensitivity analysis results indicated that none of the studies significantly influenced the combined odds ratio (OR), further confirming the validity of the study (Figures 6A,B).

### 3.6 Publication bias

**Figure 7:**
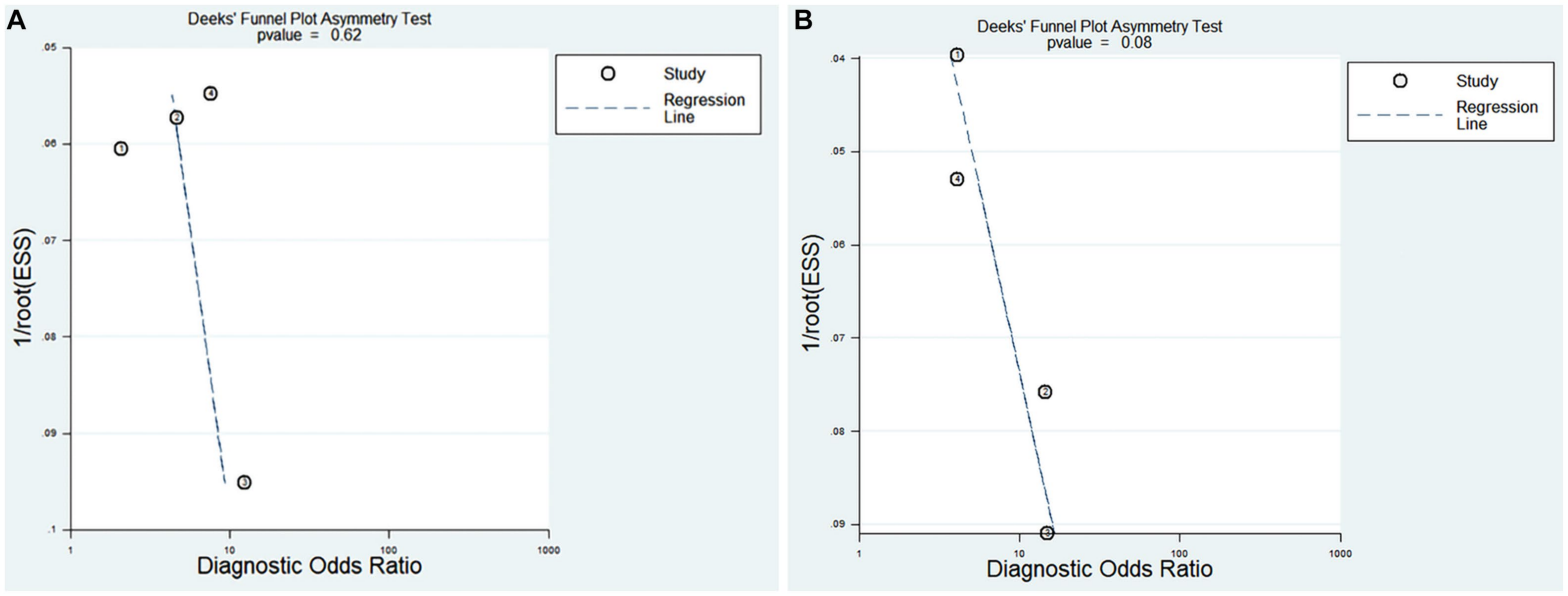
Deek’s funnel plot evaluating the potential publication bias of the included Studies (Effective Sample Size, ESS). **(A)** PNI diagnosis for KD-CAL; **(B)** PNI differentiating IVIG-resistant KD patients from IVIG-responsive KD patients.

Deek’s funnel plot asymmetry test was used to detect publication bias. As shown in Figures 7A,B, the test results indicated that no publication bias existed (*p* > 0.05).

## 4 Discussion

To our knowledge, this is the first systematic review and meta-analysis to demonstrate the diagnostic value of PNI in KD, KD-CAL, and KD with IVIG resistance. A total of 8 articles comprising 10 studies and 7,047 participants were included in this systematic review and meta-analysis. The results all indicated that PNI was identified as relatively high specificity, which indicates that PNI could be used as biomarkers for distinguish KD, KD with CAL, and KD with IVIG resistance. Sensitivity analysis conducted in this study suggested that the results were stable, and no significant publication bias was observed among the included studies. These findings suggested that PNI may serve as one of the indicators for KD diagnosis, but it is challenging to draw a definitive conclusion based on the current evidence.

The study revealed an association between low pretreatment PNI levels and a high incidence of CAL as well as IVIG resistance in KD patients, which was consistent with a previous study (18). The key variation among these studies lies in the PNI cutoff values. The range of cutoff values in the studies differentiating KD-CAL from KD-NCAL falls between 31.5 and 55.24. The range of cutoff values in the studies differentiating IVIG-resistant from IVIG-responsive KD patients fell between 47.8 and 52.4. Some studies either utilized various published thresholds from the previous literature (22) or determined their optimal cutoff values through receiver operating characteristic (ROC) analysis for their respective populations (15–17). However, due to the limited number of eligible studies, stratification of results based on cutoff values was not possible to explore which range of PNI values yields higher diagnostic efficacy. Further research is needed to explore the optimal PNI value for the diagnosis of KD-CAL and IVIG-resistant KD patients and for differentiating KD from febrile patients. The substantial interstudy heterogeneity observed in the meta-analysis should not be overlooked, although an endeavor was made to investigate the sources of heterogeneity through sensitivity analysis and publication bias analysis. However, the source of heterogeneity was not found. Therefore, caution is warranted when interpreting the results. In the future, well-designed original research with a larger sample size is needed to further explore the cutoff value for predicting KD, KD with CAL, and KD with IVIG resistance.

PNI, as a combination of serum albumin and peripheral blood lymphocytes, offers a more comprehensive reflection of the body’s nutritional and immune status (25). Since its first publication in 1983, the PNI has been employed for predicting and assessing the postoperative status of cancer patients (8). Over the past decades, malnutrition has garnered widespread attention as a prognostic indicator for various diseases, including acute myocardial infarction, heart failure, and various cancers (11, 26–30). Hence, some researchers have demonstrated that PNI may serve as a superior indicator for assessing the inflammatory and immune system status in KD patients (16, 18, 21). The PNI may provide early diagnosis and predictions for IVIG resistance in KD patients, as well as KD-CAL patients, thereby assisting in the timely implementation of appropriate treatment strategies for KD patients (16). Apparently, PNI is a simple and readily accessible diagnostic marker that can be obtained at basic health care facilities, making it cost-effective.

Currently, an increasing number of studies are emerging regarding the diagnostic and prognostic value of PNI in various diseases. A previous meta-analysis investigated the value of PNI in the prognosis of cardiovascular diseases and showed that PNI can independently predict mortality in coronary artery disease patients (31). In addition, Xiaoliang et al. have suggested that PNI may have good performance in predicting KD shock syndrome (16). Currently, research into the diagnosis and prognosis of KD is flourishing, with some investigations focused on exploring novel diagnostic markers and new predictive models. For example, our previous study (32) explored the diagnostic value of noncoding RNAs in differentiating KD-CAL from KD-NCAL, acute KD patients from convalescent KD patients, and IVIG-resistant KD from IVIG-responsive KD patients. Some studies aim to explore the diagnostic values of existing laboratory parameters in KD, such as the systemic immune-inflammation index (SII), serum ferritin and bilirubin-to-albumin (B/A) (17, 33, 34). In addition, neutrophil-to-lymphocyte ratio (NLR) and platelet-to-lymphocyte ratio (PLR) have been confirmed for their discriminative abilities to predict IVIG-resistant KD patients in previous reports (35, 36). This research indicated that PNI is at its lowest on the 6th day of disease onset, while SII is at its highest, demonstrating that both PNI and SII exhibit good predictive capabilities for KD with IVIG resistance on the 6th day of the disease course (33). The combined diagnosis of these two markers may enhance the diagnostic accuracy for KD with IVIG resistance, which is consistent with another study (16). Additionally, some studies have indicated that new predictive models have good performance in the diagnosis of IVIG-resistant KD (22, 37). The search for new potential markers that could be used in KD diagnostic models to enhance early diagnostic efficiency is ongoing. This study provides valuable clues for research on novel diagnostic markers for KD-CAL and predictive models for KD with IVIG resistance.

This study has several limitations. First, the study exhibits significant heterogeneity, possibly attributed to inconsistent study designs and variations in diagnostic criteria, resulting in clinical heterogeneity. Differences in study populations and baseline variations in treatment approaches are probably the primary reasons. Second, the study primarily included Asian countries, with a predominant focus on China, limiting the generalizability of these findings to other regions worldwide due to potential interregional and racial differences. Third, the included studies exhibited moderate to high risk of bias in patient selection and index test domains. The majority of the studies were single-center studies, and the results of multicenter studies may improve accuracy. Additionally, different PNI cutoff values would affect the consistency of the results, warranting future research to explore optimal predictive PNI cutoff values for KD, KD-CAL, and KD with IVIG resistance. Finally, this study included a total of 10 original studies, which is relatively limited. Further studies focusing on the diagnostic and prognostic values of PNI in KD are needed to provide stronger evidence.

## 5 Conclusion

In conclusion, this study was the first systematic review and meta-analysis of the diagnostic value of PNI in KD, KD-CAL, and KD with IVIG resistance. The results exhibited relatively high specificity, which indicates that PNI could be used as biomarkers for distinguish KD, KD with CAL, and KD with IVIG resistance. However, the current evidence is limited, preventing a definitive conclusion from being made. Further well-designed multicenter studies should explore its predictive value in KD, KD-CAL and KD with IVIG resistance.

## Author contributions

XZ: Writing – original draft, Conceptualization. YX: Writing – original draft, Software. HW: Formal analysis. GC: Data curation. TY: Data curation. JX: Writing – review & editing, Supervision.

## Funding

The author(s) declare that no financial support was received for the research, authorship, and/or publication of this article.
